# Supplementary figures and images for: The central histaminergic system slows visual processing in the retina and lateral geniculate nucleus of awake mice
Source: PLoS Biol. 2025 Nov 4;23(11):e3003406. doi: 10.1371/journal.pbio.3003406 (PMC12585013; doi:10.1371/journal.pbio.3003406)

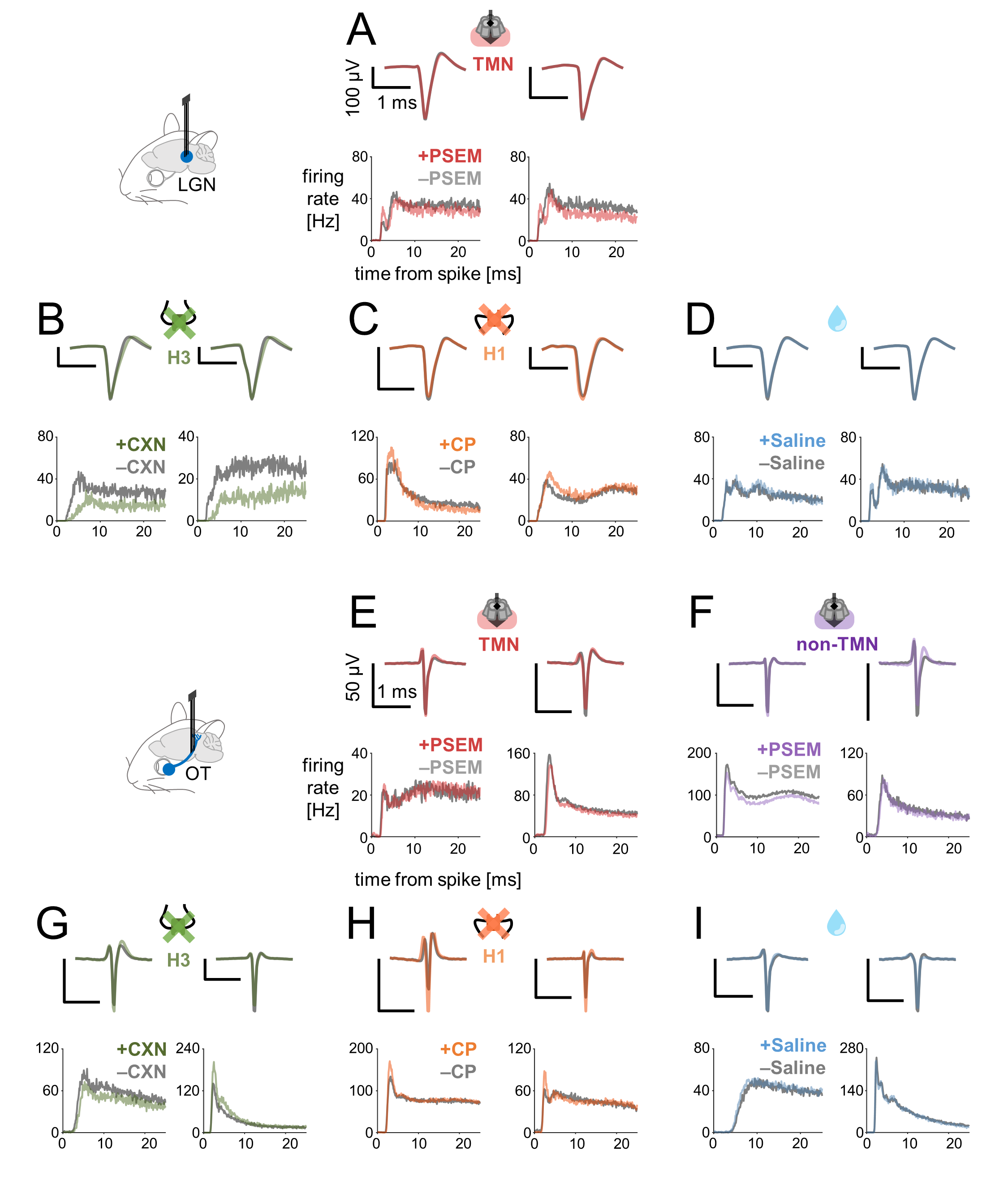

Supplement: S1 Fig — (A) Average spike waveform (top) and auto-correlogram (bottom) of two representative LGN cells (left and right) before (black) and after (red) chemogenetic activation of TMN HDC+ cells. (B–D) Corresponding data for ciproxifan (B, green), chlorphenamine (C, orange), and saline administration (D, blue), each with two representative LGN cells. (E–I) corresponding data for representative RGCs from optic tract recordings (E, chemogenetic activation of HDC+ cells in TMN; F, chemogenetic activation of HDC+ cells outside TMN; G, ciproxifan; H, chlorphenamine; I, saline). Data and code underlying this figure are available at https://doi.org/10.5281/zenodo.17016431. (TIF) [file pbio.3003406.s001.tif]

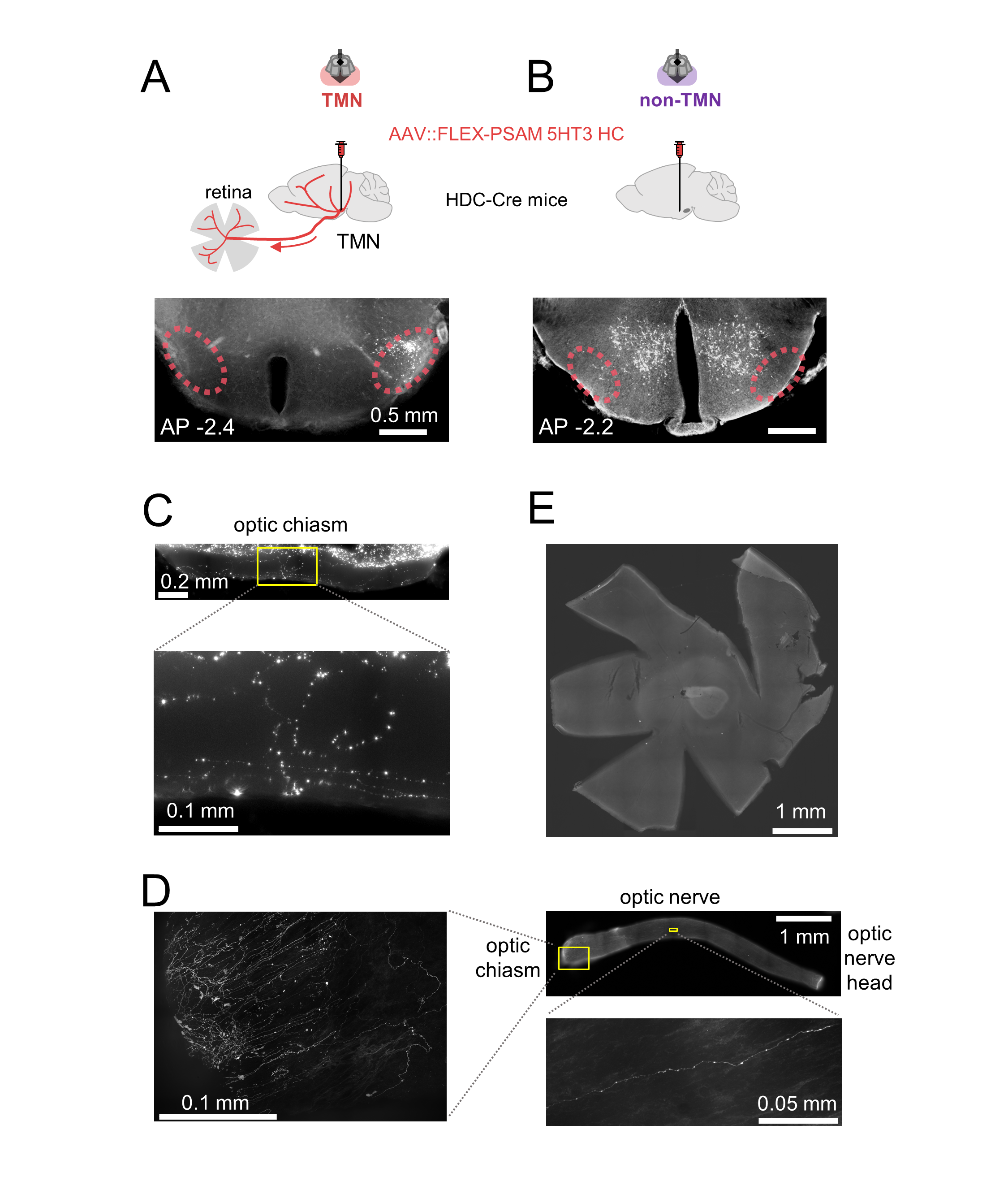

Supplement: S2 Fig — (A,B) Viral delivery of PSAM 5HT3HC channel to HDC+ cells in the TMN (marked with dotted lines) of the posterior hypothalamus (A) or those in the anterior hypothalamus (B; control). (C–E) Histological image examples of anterograde tracing of HDC+ cells in TMN. Labeled axons were found in the optic chiasm (C) or the optic nerve (D) after injecting rAAV9::CAG-FLEX-Synaptophysin-GFP or AAV2/1::CAG-FLEX-axon-GFP, respectively, in the TMN of HDC-Cre mice. However, no visible signal was detected in the isolated retinal tissue of all animals examined (n = 9; see panel E for example). Data and code underlying this figure are available at https://doi.org/10.5281/zenodo.17016431. (TIF) [file pbio.3003406.s002.tif]

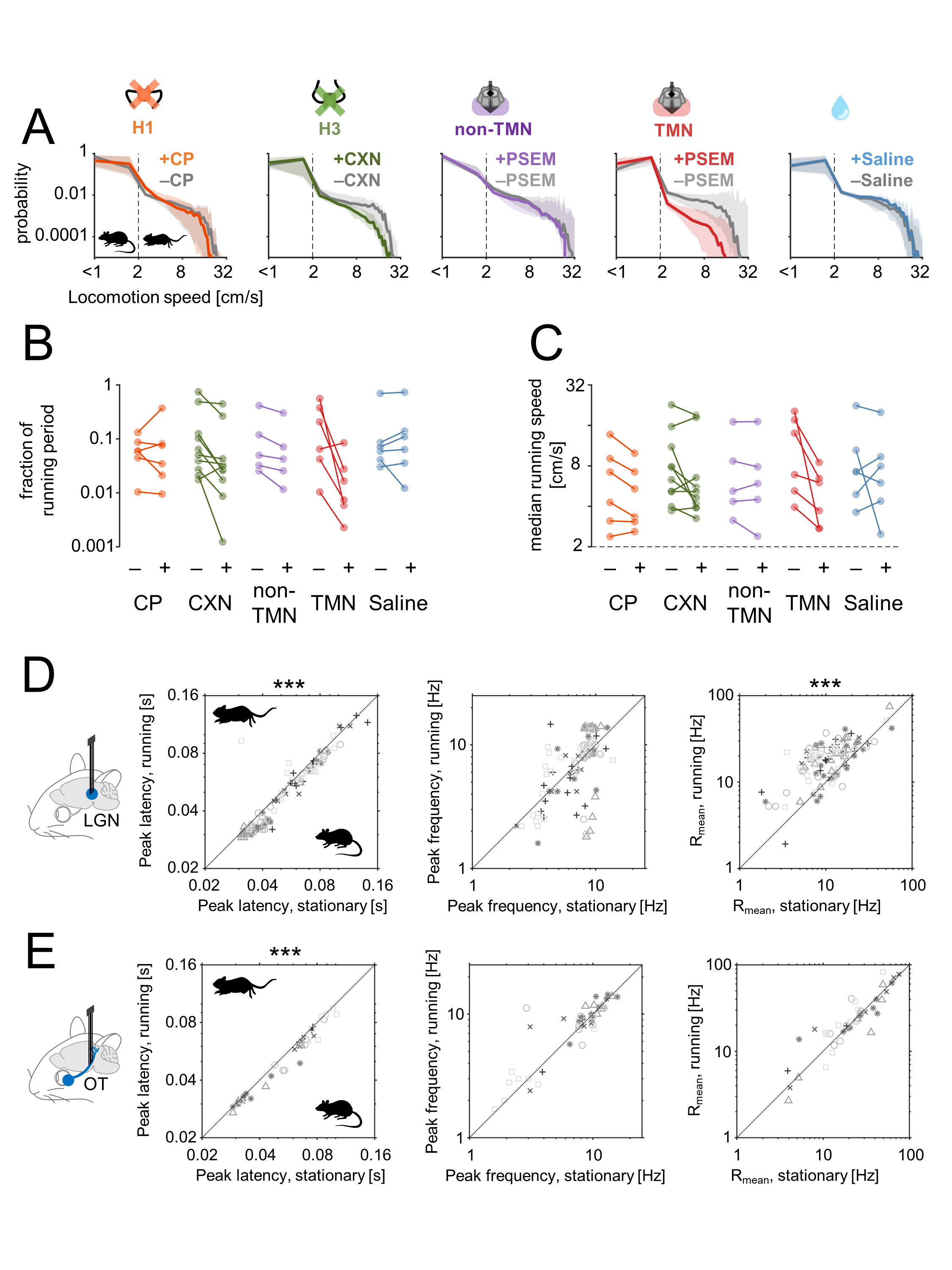

Supplement: S3 Fig — (A) Probability distribution of an animal’s locomotion speed during recordings before (gray) and after treatment: from left to right, chlorphenamine (CP, orange), ciproxifan (CXN, green), PSAM/PSEM for HDC+ cells in non-TMN (purple) or TMN (red), and saline (blue). (B) The fraction of running period (>2 cm/s) before and after each treatment. No significant change was observed (p = 0.07, Kruskal-Wallis test). (C) The median locomotion speed (during which animals moved at >2 cm/s) before and after each treatment. No significant change was observed (p = 0.3). (D) Comparison of the LGN population response properties between stationary and running periods (n = 104 from 5 animals with a running period ranging between 20% and 80%): from left to right, peak latency (50 ± 19 ms versus 48 ± 19 ms, median ± median absolute deviation; p < 0.001, Wilcoxon signed-rank test), peak frequency (8.2 ± 1.9 Hz versus 8.2 ± 3.0 Hz; p = 0.4), mean firing rate (11 ± 6 Hz versus 20 ± 8 Hz; p < 0.001). (E) Corresponding data for RGCs (n = 44 from 5 animals with a running period ranging between 20% and 80%): from left to right, peak latency (58 ± 19 ms versus 49 ± 18 ms; p < 0.001), peak frequency (8.2 ± 2.8 Hz versus 9.2 ± 2.7 Hz; p = 0.6), mean firing rate (24 ± 22 Hz versus 23 ± 22 Hz; p = 0.5). Data and code underlying this figure are available at https://doi.org/10.5281/zenodo.17016431. (TIF) [file pbio.3003406.s003.tif]

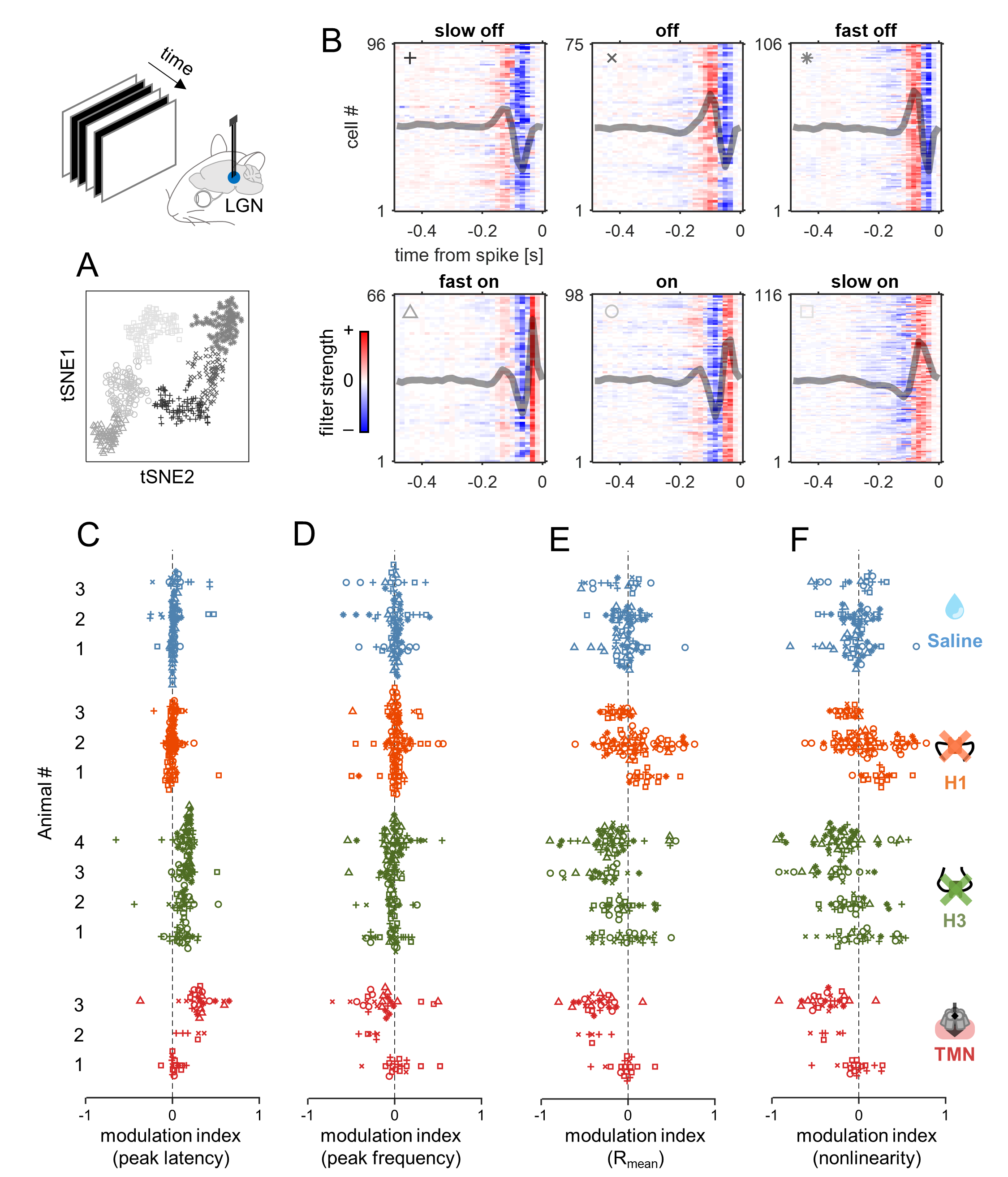

Supplement: S4 Fig — (A) t-Distributed Stochastic Neighbor Embedding (t-SNE) embedding of the STAs of LGN cells. Different markers and shadings are used for distinct response categories (shown in B). (B) Each panel represents one of the six response categories: slow off, off, fast off, fast on, on, and slow on. In each panel, each row represents a cell’s STA (color-coded with red and blue hue, indicating positive and negative filter values, respectively); and the overlaid gray line shows the average STAs in each response type. (C–F) Modulation indices on LGN response characteristics across individual animals: from left to right, peak latency (C), peak frequency (D), mean evoked firing rate (E), and nonlinearity (F). The effects of histamine were largely consistent across animals. Data and code underlying this figure are available at https://doi.org/10.5281/zenodo.17016431. (TIF) [file pbio.3003406.s004.tif]

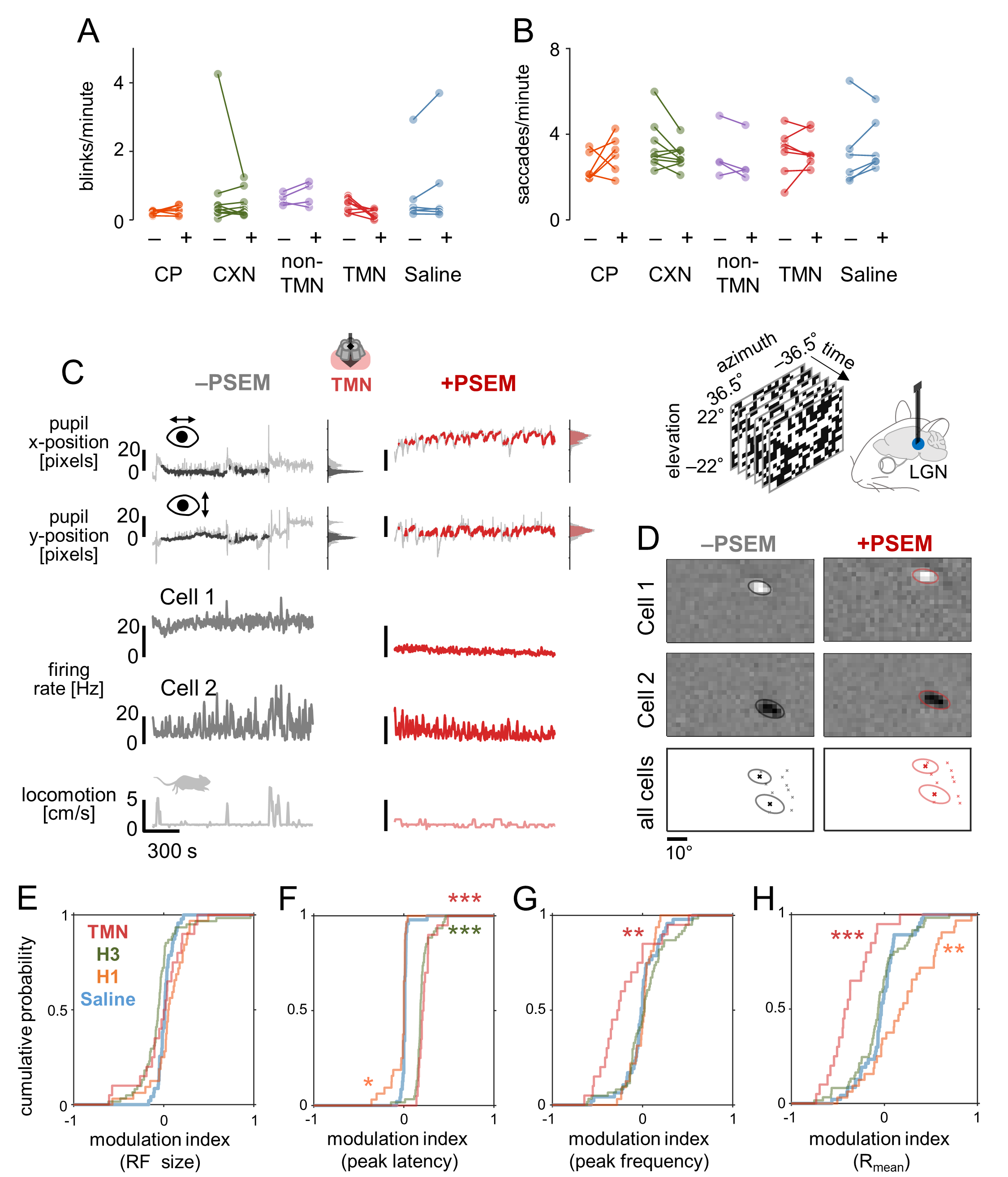

Supplement: S5 Fig — (A, B) Blink (A) and saccade (B) frequencies before and after different treatments: from left to right, chlorphenamine (CP), ciproxifan (CPN), PSAM/PSEM for non-TMN HDC+ cells and TMN HDC+ cells, and saline. None of these changes were statistically significant: p = 0.10, Kruskal–Wallis test on blink frequency changes; p = 0.15, saccade frequency changes. (C) Representative time series of the eye position (X- and Y-coordinates of the pupil center extracted from eye-tracking camera images) during white-noise “checkerboard” stimulation (black/red, centered and stable eye position used for reverse correlation; gray, non-centered or non-stable period excluded from the analysis; probability distribution shown on the right), before (left) and after (right) chemogenetic activation of TMN HDC+ cells. (D) Estimated spatial filter (receptive field; RF) of two example LGN cells before (top) and after (bottom) chemogenetic activation of HDC+ cells in TMN. Note a shift of RF position of all recorded cells due to a shift of the animal’s resting eye position after the treatment (see panel C). (E–H) Cumulative distribution of the modulation index of LGN cells before and after treatment (E, receptive field size; F, peak latency; G, peak frequency; H, mean evoked firing rate): orange, chlorphenamine, n = 32 from 3 animals; green, ciproxifan, n = 61 from 4 animals; red, PSAM/PSEM for HDC+ cells in TMN, n = 20 cells from 3 animals. Post-hoc test against saline control (n = 47 from 3 animals) after Kruskal–Wallis test: * p < 0.05; ** p < 0.01; *** p < 0.001. Data and code underlying this figure are available at https://doi.org/10.5281/zenodo.17016431. (TIF) [file pbio.3003406.s005.tif]

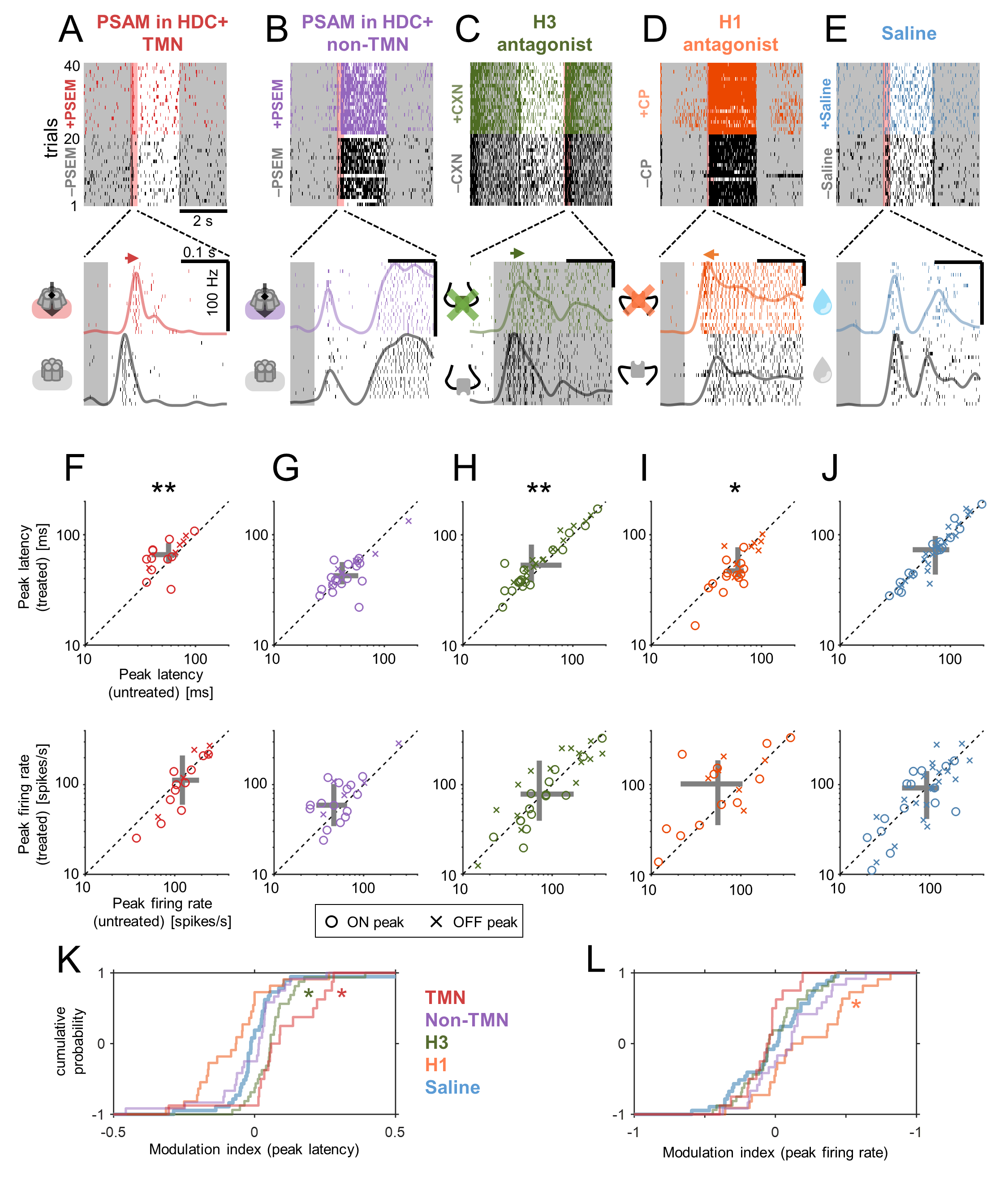

Supplement: S6 Fig — (A–E) Visual responses of a representative RGC to full-field contrast-inverting stimuli (2 s intervals) before and after chemogenetic (A, HDC+ cells in TMN; B, HDC+ cells in non-TMN) or pharmacological treatment (C, ciproxifan; D, chlorphenamine; E, saline); top, spike raster across trials; bottom, zoom-in of the spike raster around stimulus onset or offset (−50 to 250 ms, red shade on top) and peri-stimulus time histogram. (F–J) Pairwise comparison of the RGC population responses before and after treatment (top, peak latency; bottom, peak firing rate): * p < 0.05; ** p < 0.01, Wilcoxon signed-rank test with Bonferroni correction. F, PSAM/PSEM for TMN HDC+ cells: 57 ± 15 ms versus 66 ± 17 ms peak latency, p = 0.005; 122 ± 54 Hz versus 112 ± 66 Hz peak frequency, p = 0.08; median ± median absolute deviation, n = 16 RGCs from 4 animals. G, PSAM/PSEM for non-TMN HDC+ cells: 42 ± 17 ms versus 43 ± 14 ms peak latency, p = 0.3; 46 ± 30 Hz versus 59 ± 40 Hz peak frequency, p = 0.3; n = 24 RGCs from 3 animals. H, ciproxifan: 42 ± 31 ms versus 53 ± 31 ms peak latency, p = 0.002; 72 ± 80 Hz versus 78 ± 78 Hz peak frequency, p = 0.3; n = 32 RGCs from 4 animals. I, chlorphenamine: 61 ± 16 ms versus 47 ± 18 ms peak latency, p = 0.026; 55 ± 60 Hz versus 102 ± 88 Hz peak frequency, p = 0.05; n = 22 RGCs from 3 animals. J, saline: 73 ± 30 ms versus 73 ± 31 ms peak latency, p = 0.5; 92 ± 50 Hz versus 92 ± 56 Hz peak frequency, p = 1; n = 37 RGCs from 3 animals. (K,L) Cumulative distributions of the modulation index before and after each treatment (in corresponding colors): K, peak latencies; L, peak firing rate; * p < 0.05 from the post-hoc test against the saline (control) condition on the average group ranks (Kruskal–Wallis test). Data and code underlying this figure are available at https://doi.org/10.5281/zenodo.17016431. (TIF) [file pbio.3003406.s006.tif]

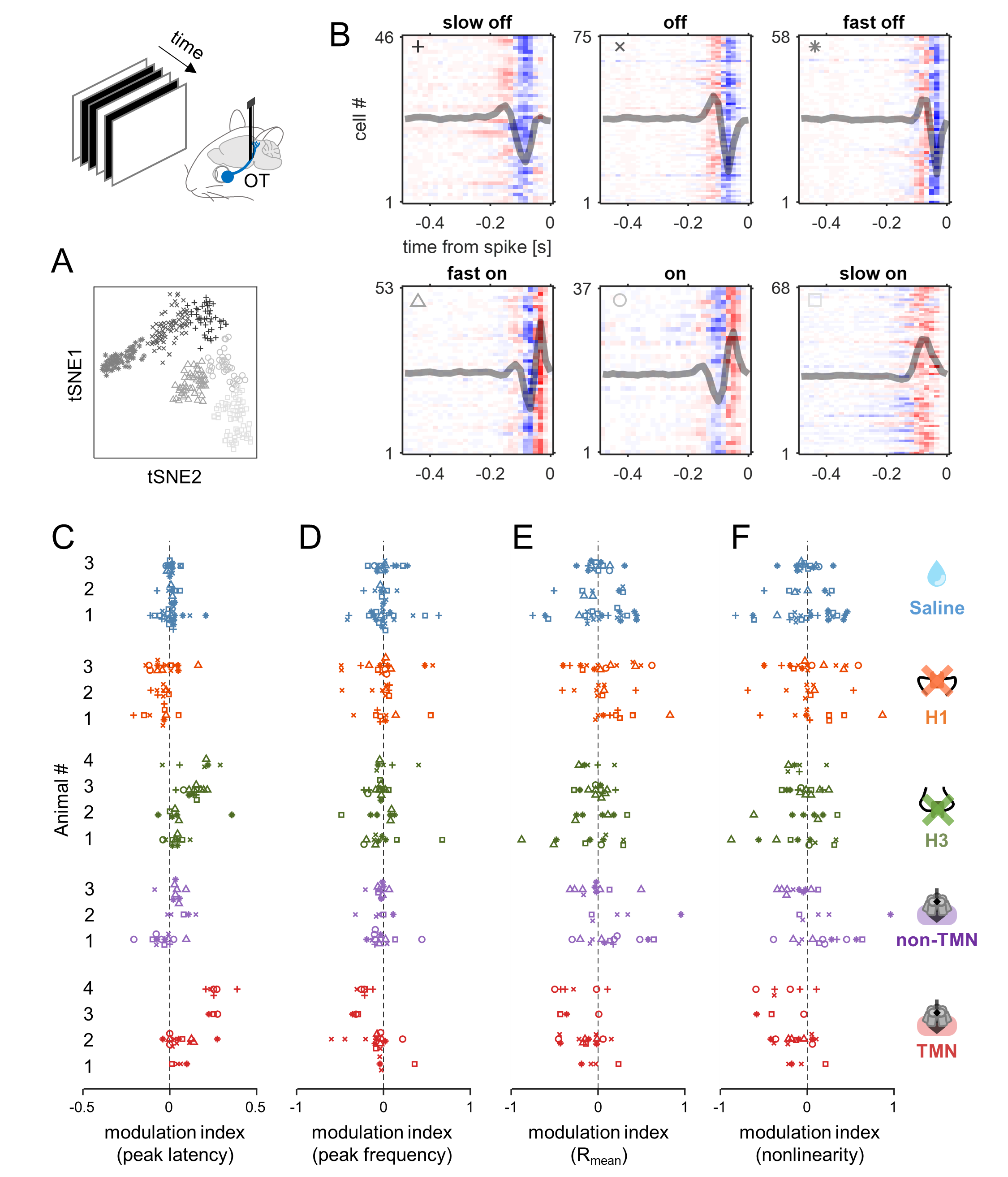

Supplement: S7 Fig — (A) t-SNE embedding of the RGC STAs. Different markers and shadings are used for distinct response categories (shown in B). (B) Each panel represents one of the six response categories: slow off, off, fast off, fast on, on, and slow on. In each panel, each row represents a cell’s STA (color-coded with red and blue hue, indicating positive and negative filter values, respectively); and the overlaid gray line shows the average STAs in each response type. (C–F) Modulation indices on RGC response characteristics across animals: from left to right, peak latency (C), peak frequency (D), mean firing rate (E), and nonlinearity (F). The effects of histamine were generally consistent across animals, and no substantial batch effect was observed. Data and code underlying this figure are available at https://doi.org/10.5281/zenodo.17016431. (TIF) [file pbio.3003406.s007.tif]

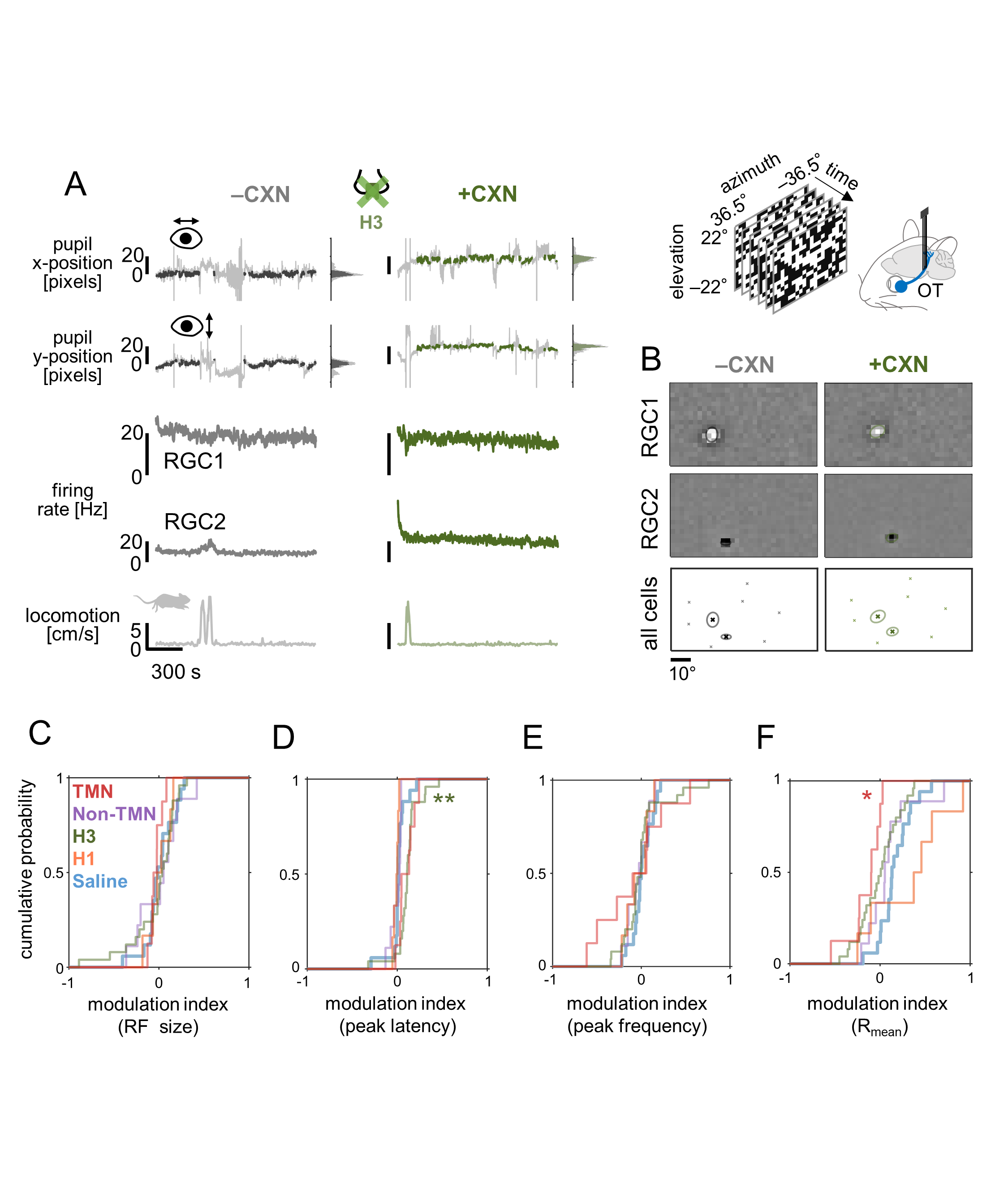

Supplement: S8 Fig — (A) Representative time series of the eye position (X- and Y-coordinate of the pupil center extracted from eye-tracking camera images) during white-noise “checkerboard” stimulus presentation (black/green, centered and stable eye position used for reverse correlation analysis; gray, non-centered or non-stable period excluded from the analysis; probability distribution shown on the right), along with those of firing rate dynamics of two example RGCs and locomotion (from top to bottom), before (left) and after (right) ciproxifan administration. (B) Estimated spatial filter (receptive field) of the two example RGCs (top and middle) before (left) and after (right) ciproxifan administration. Note a shift of RF position of all recorded cells due to a shift of resting eye position after the treatment (see panel A). (C–F) Cumulative distribution of the modulation index of RGCs before and after treatment (C, receptive field size; D, peak latency; E, peak frequency, F, mean firing rate): orange, chlorphenamine, n = 6 cells from 3 animals; green, ciproxifan, n = 25 cells from 4 animals; purple, PSAM/PSEM for HDC+ cells in non-TMN, n = 9 cells from 3 animals; red, PSAM/PSEM for HDC+ cells in TMN, n = 8 cells from 4 animals. Post-hoc test against saline control (n = 17 from 3 animals) after Kruskal–Wallis test: * p < 0.05; ** p < 0.01. Data and code underlying this figure are available at https://doi.org/10.5281/zenodo.17016431. (TIF) [file pbio.3003406.s008.tif]

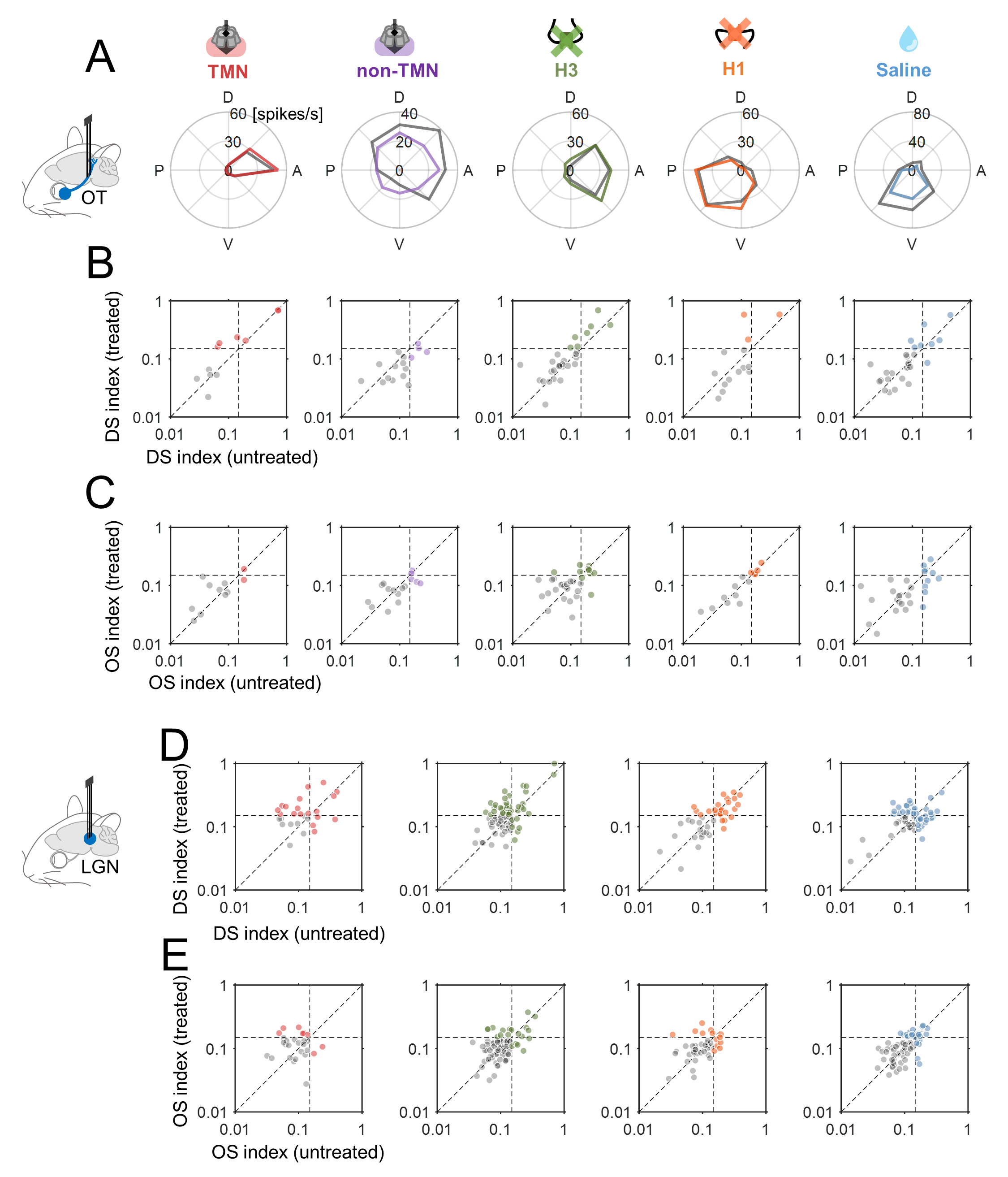

Supplement: S9 Fig — (A) The average firing rate of a representative direction-selective (DS) RGC in response to moving gratings in eight different directions before (gray) and after treatment (color-coded): from left to right, PSAM/PSEM for TMN HDC+ cells (red) or non-TMN cells (purple), ciproxifan (green), chlorphenamine (orange), and saline (blue). (B) DS indices of RGC populations before and after treatment. Those with DS index >0.15 in either condition were highlighted in the corresponding color (from left to right, n = 11, 16, 31, 14, and 30 RGCs, respectively). No significant change was observed in the DS index values: p > 0.2 in all cases (Wilcoxon signed-rank test). (C) Corresponding data for the OS indices of RGCs. No significant change was observed (p > 0.7 in all cases). (D,E) Corresponding population data for the DS and OS indices of LGN cells (from left to right, n = 42, 76, 27, 58 LGN cells; p > 0.06 and 0.3 in all cases, respectively). Data and code underlying this figure are available at https://doi.org/10.5281/zenodo.17016431. (TIF) [file pbio.3003406.s009.tif]

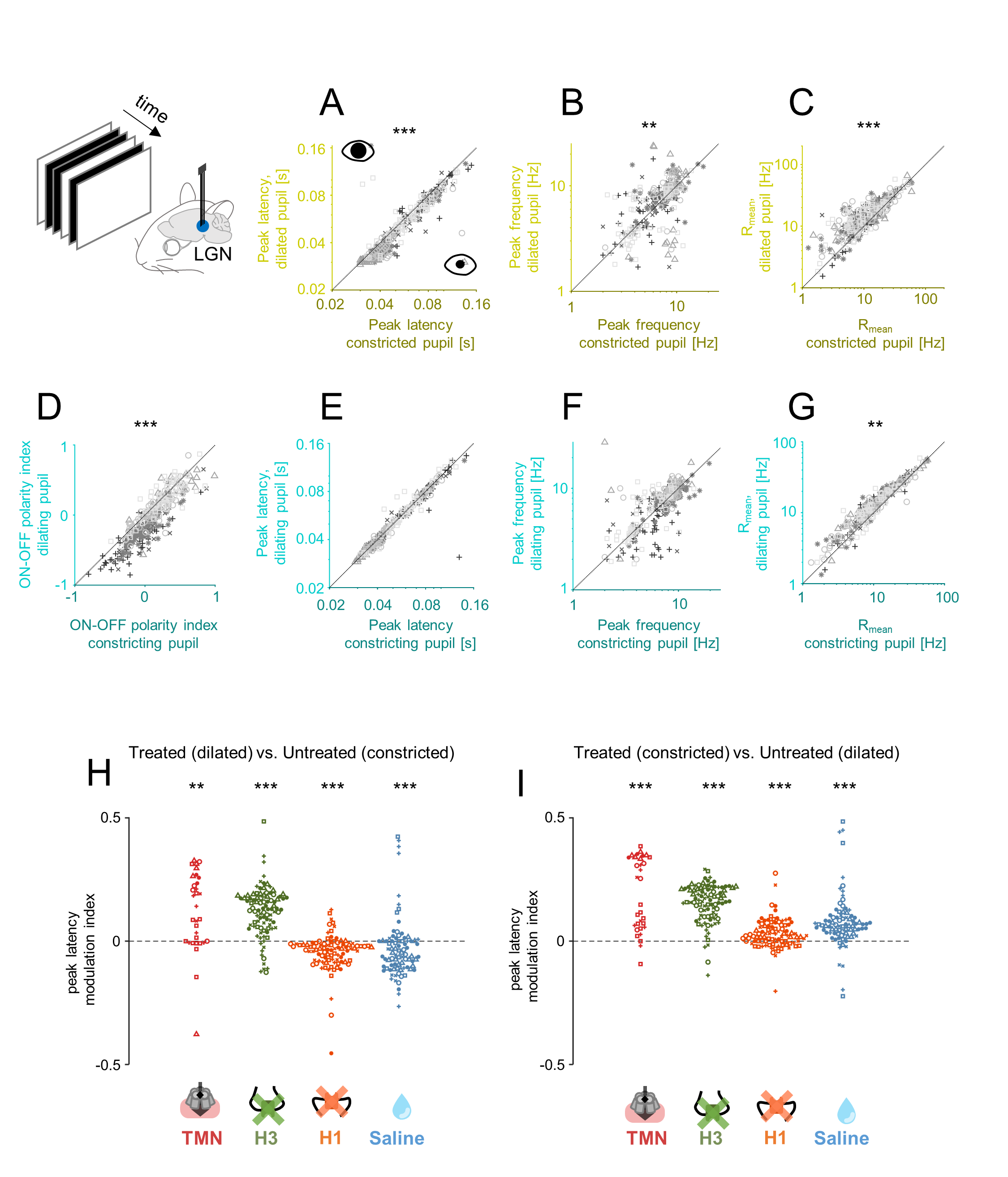

Supplement: S10 Fig — (A–C) Comparison of the LGN population response properties between constricted and dilated pupil periods (n = 350 RGCs from 13 animals): A, peak latency, 51 ± 20 ms versus 48 ± 19 ms, median ± median absolute deviation, p < 0.001, Wilcoxon signed-rank test; B, peak frequency, 7.9 ± 2.1 Hz versus 8.1 ± 2.4 Hz, p = 0.004; C, mean firing rate, 11 ± 7 versus 15 ± 7 Hz, p < 0.001. (D–G) Comparison of the LGN population response properties between constricting and dilating pupil periods: D, ON-OFF polarity index of the temporal filters, 0.07 ± 0.22 versus −0.06 ± 0.27, p < 0.001; E, peak latency, 49 ± 19 ms versus 49 ± 19 ms, p = 0.8; F, peak frequency, 7.9 ± 2.0 Hz versus 8.1 ± 2.1 Hz, p = 0.3; G, mean firing rate, 11 ± 7 Hz versus 14 ± 7 Hz, p = 0.004. (H) Comparison of peak latencies before treatment with constricted pupil versus after treatment with dilated pupil: from left to right, PSAM/PSEM for TMN HDC+ cells (red), ciproxifan (green), chlorphenamine (orange), and saline (blue): ** p < 0.01; *** p < 0.001, Wilcoxon signed rank test. (I) Comparison of peak latencies before treatment with dilated pupil versus after treatment with constricted pupil. Data and code underlying this figure are available at https://doi.org/10.5281/zenodo.17016431. (TIF) [file pbio.3003406.s010.tif]
