# Supplementary material for: The central histaminergic system slows visual processing in the retina and lateral geniculate nucleus of awake mice
Source: PLoS Biol. 2025 Nov 4;23(11):e3003406. doi: 10.1371/journal.pbio.3003406 (PMC12585013; doi:10.1371/journal.pbio.3003406)
Supplement: Attachment — Submitted filename: Response_to_reviewers.pdf [file pbio.3003406.s013.pdf]

We thank the reviewers for their constructive comments (in blue). Below are our point-by-point replies (in black).

Reviewer #1:

In this manuscript, Tripodi and Asari investigate the effects of histamine on early visual processing in mice. They do so by combining (i) awake silicon probe recordings placed in the dLGN or optic tract, (ii) visual stimuli, (iii) a combination of pharmacological interventions to activate or suppress histaminergic receptors, (iv) pharmacogenetic perturbations (PSAM/PSEM), and (v) modeling. Their results and conclusions suggest that histamine release from the hypothalamic tuberomammillary nucleus modulates the rate and response kinetics of visual stimuli. These adaptations may have implications for state-dependent visuomotor processing.

The paper is well-written and presented, and the results are convincing. I have only a few major and minor points to discuss, which I believe need to be address prior to publication.

Major:

1. My main concern is behavioral control, which as the authors mention, could account for the observed effects. Thus, I would kindly ask for a more detailed behavioral analysis, not just the average but the distributions across animals. Let me be a bit more specific:

One of the main arguments goes: When applying H1 antagonist (mimicking a decrease of histamine), the pupil is dilated, using an H3 antagonist (mimicking an increase of histamine), the pupil is constricted. Counter to these pharmacological results, increasing histamine release using a chemogenetic approach has the opposite effect, constricting the pupil. These contrary results are taken as proof that the changes in the temporal dynamics are not related to luminance (pupile size changes). However, to be truly convincing, it would be necessary to show the dynamics of constriction with respect to the time the stimulus was recorded and the distribution of pupil sizes. The authors only show the relative size changes, but it is well possible that due to the manipulations, these start at a different baseline, are changed for a short period, or show different constriction dynamics. Thus, showing distributions with the absolute pupil sizes during the recording period would be more informative and compare the sizes during the recordings. For example, the authors could subsample times of similar pupil size to estimate the temporal filters. Ideally, one would use atropine to dilate the pupil together with the pharmacological perturbations. That would completely eliminate any luminance change and the difference can be directly attributed to the modulation histamine (perhaps even enhancing them), but a more detailed analysis of the behaviour might be sufficient.

We thank the reviewer for the suggestions. Just to be clear, on average we found pupil dilation, not constriction, upon chemogenetic activation of HDC+ neurons (increasing histamine) or H1 antagonist application (decreasing histamine), while they had opposite effects on retinal ganglion cell (RGC) dynamics. Moreover, H3 antagonist application (increasing histamine) led to pupil constriction, while it had the same effect on RGC dynamics as the chemogenetic manipulation. These led us to conclude that the overall pupil size cannot explain our experimental results on the retinal dynamics. To further strengthen our conclusion, we further analyzed the effects of the pupil size and dynamics in more detail.

a. Pupil size effects

There was a considerable variability in the baseline pupil size across animals. We thus analyzed the data using relative rather than absolute pupil size. In particular, we subsampled the data for each recording based on the pupil size (constricted, below 33.3 percentile; neutral, between 33.3 to 66.7 percentile; dilated, above 66.7 percentile; 100 second each in total), and estimated the RGC temporal filters in each period (**Figure 4** in the revised manuscript; see **Supplementary Figure 10** for LGN cells). We then found that <u>the larger the pupil size, the faster the RGC kinetics in general (**Figure 4D**)</u>.

As reported before (Fang et al., 1998), chlorphenamine (H1 antagonist) led to a substantial increase in the baseline pupil size due to its anticholinergic properties (**Figure 4K**). We found that RGC kinetics after blocking H1 with relatively small pupil sizes were not significantly different from those under the control condition with relatively large pupil sizes (**Figure 4M**). Thus, here we cannot exclude a possible contribution of the baseline pupil dilation on the response facilitation effect of chlorphenamine.

In contrast, ciproxifan (H3 antagonist) led to a slight decrease in the baseline pupil size, while chemogenetic activation of HDC+ cells led to a slight increase, though they both resulted in slower RGC response dynamics. Importantly, here we found that RGC kinetics after those manipulations with relatively large pupil sizes were nevertheless significantly slower than those under the control condition with relatively small pupil sizes (**Figure 4L**). Thus, the resulting changes in the RGC response dynamics after these treatments cannot be attributable to the pupil size effect.

b. Pupil dynamics effects

Changes in pupil size are much slower than the RGC response dynamics: e.g., pupillary light reflex has a latency of hundreds of milliseconds and a time-constant of about a second (Grozdanic et al., 2009; Liu et al., 2017). Nevertheless, when the pupil is dilating, presented stimuli on the screen should appear brighter than intended, thereby biasing RGC responses toward darker stimuli, whereas one would expect the opposite effect when the pupil is constricting. We indeed identified such <u>changes in the ON/OFF balance of the estimated temporal filters</u> (**Figure 4G**), but found <u>no change in their response kinetics</u> (**Figure 4H**). Therefore, the effects of the pupil dynamics should have little to do with the observed changes in the response kinetics after the pharmacological or chemogenetic manipulations. This further supports our claim that histamine slows down the RGC response dynamics.

2. It would also be relevant to take a look at the eye movements. Are the position of the eyes changes, do the authors see more saccades? One indication of this is the presented RF in Supplementary Fig. 3. While the size of the RF changes, it seems that the position might also change. Could that be accounted for by more or less eye movements?

We found no substantial changes in the frequencies of saccades or eye blinks after the pharmacological or chemogenetic treatments (**Supplementary Figure 5A,B**). In contrast, the baseline eye position often changed between the two recordings (e.g., **Supplementary Figures 5C and 8A**), which led to a shift of the receptive field (RF) position in all the simultaneously recorded cells while maintaining their topographic arrangement (e.g., **Supplementary Figures 5D and 8B**).

To better estimate the RF size, we reran the RF analysis using subsampled data where the eye position was centered and stable. More specifically, we included only those data within an interquartile

range for at least 2 seconds to minimize artefacts due to eye movements (while retaining enough data for RF estimation). We then obtained more confined – often less elongated – receptive fields in general, and found that the RF size was not significantly affected in either LGN (**Supplementary Figure 5**) or RGCs (**Supplementary Figure 8**).

3. Regarding the locomotion speeds, the authors also use a very low threshold for "running" >2 cm/s. Moreover, only 20% of the time animals were "running". Taking the mean of many low entries is necessarily very low, this the ratio of treated and untreated, as plotted in Fig. 3B, C, might not be the best way to present the results. I suggest that the authors should compare the distributions of absolute running speeds, and make sure that the periods used to characterize the response properties have similar behavioral properties (excluding running periods from the analysis, e.g.).

Spontaneous running behavior varied a lot between animals, or even substantially between the two recordings of the same animal before and after saline injection. Nevertheless, animals tend to run slower after the manipulations, especially after chemogenetically activating HDC+ cells in TMN (**Supplementary Figure 3A**), though we found no significant difference between the control and drug conditions in the fraction of the running period (**Supplementary Figure 3B**) or the median speed during the running period (i.e., excluding the time when animals were moving less than 2 cm/s; **Supplementary Figure 3C**).

To clarify the effects of an animal's behavior on RGC dynamics, we performed the reverse-correlation analysis in stationary and running periods (at a threshold of 2 cm/s), respectively (**Supplementary Figure 3D,E**). We then found that <u>RGC/LGN response kinetics were generally faster during the running period than the stationary period</u>. We thus excluded the data during the running period when we compared the visual response properties before and after treatments (**Figures 2 and 3**), and obtained the results consistent with our original claim that histamine slows down the RGC/LGN response dynamics.

4. While the temporal filters are compelling, this is an indirect description of visual responses. Would the authors have examples of the same cell to repetitions of the same stimulus (e.g., full-field flashes) with different pharmacological perturbations? Perhaps the authors could find instances during their WN stimulus of identical changes and present them as a raster plot? That would provide a direct link between the gain (changes in rate) and kinetics (speeds of the first spike) of the responses.

We examined the responses to full-field contrast-inverting stimuli (2 s ON / 2 s OFF), and found the same effects on the peak latencies for both ON and OFF responses in both RGCs and LGN cells (**Figures 1 and Supplementary Figure 6**). This further supports our conclusion about the histaminergic modulation of the mouse early visual system. Note, however, that here we were not able to control the animal's behavior or pupil dynamics as we have only 20 trials in each recording condition. In contrast, we had long enough data to estimate temporal filters at different behavioral states (**Figure 4 and Supplementary Figures 3 and 10**). Thus, the analysis based on the temporal filters is critical to support our claim.

5. The statistical quantification in Figs. 1 and 2 are for the entire population. It would be good to compare all cells recorded for each animal. I would appreciate it if the authors could plot the distributions for all cells recorded for each animal as violin plots with each cell represented.

We examined the data for each animal, and found consistent results across animals (**Supplementary Figures 4C-F and 7C-F**). There was thus little batch effect.

6. Finally, I'm curious about histaminergic projections to the retina. These projections have been reported recently and cited by the authors. I have heard, however, that the histaminergic projections to the retina are often not visible. I wonder if the authors see these projections in retinal explants. Either result is interesting since there are clear physiological effects. I wonder if they don't see them, if histamine could modulate retinal ganglion cell axons in the brain by changing the propagation of action potentials or synaptic release. I would be interested to know what the authors think.

Here we failed to detect any labelled axons of TMN HDC+ cells in the retina (**Supplementary Figure 2E**). However, a) we detected these axons in the optic chiasm and the optic nerve (3 out of 10 animals; **Supplementary Figure 2C,D**); b) we used the same mouse lines as previous studies that demonstrated the presence of histaminergic / HDC+ axons in the retina (Greferath et al., 2009; Warwick et al., 2024); and c) we observed the modulation at the level of RGC axons in the optic tract. These results support that the modulation arises in the retina.

Axon potentials should have reached the recording site in the optic tract within 1 ms after exiting the eye cup, given the optic nerve's conduction velocity at ~10 m/s (Sumitomo et al., 1969) and its length below 10 mm in mice. While the axon conduction velocity depends on various factors, including myelination, G-ratio, axon diameter, and sodium channel density, modulation at the level of axon conduction velocity should be on the order of sub-milliseconds (Etxeberria et al., 2016), hence cannot explain our data.

Minor:

Title:

- I suggest changing the title to be more specific. "Interferes" is correct, but does not convey the essence of the finding. Knowing that gain and kinetics are modulated would attract more readers.

We now revised the title of our manuscript as follows: "Central histaminergic system reduces response gain and slows visual processing in the retina and lateral geniculate nucleus of awake mice."

Abstract:

- Second sentence. Please change "even at the retina" to "already".

Done.

- "Our computational modeling analysis instead suggests neural circuit effects." Given that the previous sentence is about behavior, I would suggest for readability that you clearly define what the modeling suggests. Something like: "Instead, our computational modeling analysis suggests that the temporal response modulations are due to changes in the intrinsic properties of the circuit."

We have revised the abstract, following the reviewer's suggestion.

Main text:
Line 229. The sentence "we cannot deny" seems strange. I would suggest changing it to something of the sort: "Thus, it is possible tat the observed hitaminergic effects on the visual response properties in the muse early visual system are a result of correlated behaviorla modulation."

We have revised the text.

Reviewer #2:

Based on in vivo electrophysiological recordings of visual responses in the optic tract and dLGN of awake head-fixed mice, both with and without pharmacological and chemogenetic manipulations, Tripodi et al. concluded that the early visual system, including retinal ganglion cells (RGCs) and dLGN neurons, exhibited reduced and slowed visual responses when histaminergic neurons in the tuberomammillary nucleus (TMN) of the posterior hypothalamus were activated. Furthermore, the effects of histaminergic modulation could not be attributed to changes in pupil dynamics or locomotion behavior. Instead, their computational modeling suggests that histaminergic modulation of the early visual system occurs via histamine H1 receptors in the retina. These findings are surprising, as high histamine levels are typically associated with high vigilance, yet here, high histamine resulted in weaker and slower responses in the early visual system, contrary to expectations.

Histaminergic modulation of early visual processing could be a significant topic in neuroscience. However, I have several major concerns about the experimental setup and results that need to be addressed to support and validate the authors' conclusions.

Major comments:

1. This manuscript was based on electrophysiology recordings but did not describe the spike sorting method used, nor whether single-unit signals were reliably isolated from their recordings.

Here we adapted previously established methods of spiking sorting (Boissonnet et al., 2023). In brief, we first concatenated the raw recording data before and after treatments into a single binary file, and used Kilosort 2.0 to sort spikes with a set of default parameters, except for the spike detection threshold to be 6 during optimization. Single-units were then identified by clustering in principal component space using Phy for manual data curation. Only those units that maintained the average spike waveforms and auto-correlograms with a minimal refractory period of 1 ms were kept for subsequent analyses (**Supplementary Figure 1**).

2. It is unclear whether the comparison before and after chemogenetic or pharmacological manipulation was performed from the same single units. If so, evidence should be provided to demonstrate that the recordings from the same single units (especially from the optic tract) were stable over time.

The comparison was done from the same single units throughout the manuscript. To clarify this, the revised manuscript shows spike waveforms and auto-correlograms before and after treatments for representative units in each recording condition (**Supplementary Figure 1**). We will also make all the data and code publicly available upon acceptance for publication.

3. The methods section did not specify where the probe was implanted in the optic tract or provide images of the implanted area. This lack of detail makes it impossible to rule out the possibility that the recorded signals from the optic tract originate from axons of neuron types other than retinal ganglion cells. For example, axons from the parabigeminal nucleus (PBG) follow the optic tract to project to the contralateral dLGN ('PBG axons coursed within the ipsilateral SOX toward the optic chiasm, crossing the ventral midline, and running dorsally within the optic tract en route to dLGN', Sokhadze et al., J Comp Neurol. 2021; see also Figure 1 in Tokuoka et al., J Neurophysiol 2020). Moreover, not all the retinal axons in the optic tract project to the dLGN.

We performed optic tract recordings in awake head-fixed mice as described previously (Boissonnet et al., 2023). Specifically, electrodes were inserted into [-1.34, +1.87, +4.74], [-1.70, +1.87, +4.74], or [-1.82, +2.35, +4.07] in [Anterior-Posterior, Medial-Lateral, Dorsal-Ventral] coordinates using a robotic stereotaxic system. We then selected RGCs as those with robust visual responses: i.e., $SNR = \mathrm{var}[\mathrm{mean}[r(t)]_{\mathrm{trials}}]_{\mathrm{time}}/\mathrm{mean}[\mathrm{var}[r(t)]_{\mathrm{time}}]_{\mathrm{trials}} > 0.15$, where $r(t)$ is the response to full-field contrast inverting stimuli at maximum contrast; and the estimated temporal filter should have $p < 10^{-10}$ in at least one time bin within 200 ms from the spike onset. These units typically had a triphasic spike waveform as expected for axonal signals (Barry, 2015). In contrast, single-units with little or no visual responses were considered as non-RGCs and excluded from the analysis in this study. These non-RGCs may include PBG cells as they have axons in the optic tract (Tokuoka et al., 2020), but no robust visual responses (Reinhard et al., 2019). We clarified these points in the revised methods section, and also included representative histology data to show the probe position in the optic tract (**Figure 3B**) or LGN (**Figure 1B**).

4. This study suggests that the effects of chemogenetic activation of histaminergic neurons in the TMN on the early visual system are 'direct,' mediated by histamine receptors in the retina, rather than indirectly through modulating arousal or locomotion states. However, no evidence is provided to demonstrate that their TMN injection labeled any histaminergic axon terminals in the retina. This is especially concerning given that the number of labeled TMN neurons appears to be small (Figure S1), and histaminergic axons in the retina are known to be very sparse according to the literature.

As described above (reviewer #1, major item #6), we failed to detect labelled axons of TMN HDC+ cells in the retina (e.g., **Supplementary Figure 2E**). However, a) we detected these labelled axons in the optic chiasm/nerve (3 out of 10 animals; **Supplementary Figure 2C,D**); b) we used the same mouse lines as previous studies that demonstrated the presence of histaminergic / HDC+ axons in

the retina (Greferath et al., 2009; Warwick et al., 2024); and c) we observed the modulation at the level of RGC axons in the optic tract. These results support that the modulation arises in the retina.

It should also be noted that histamine can modulate neuronal activity over a large area, rather than just at the release site. Histamine is a relatively small molecule with a low net charge, allowing it to travel a long distance from the release site and diffuse widely in the brain tissue. This is likely why sparse projection is enough for histamine to serve as a volume transmitter across the brain, including the retina; and also, why we (and many others) failed to detect these axons (Repérant et al., 2006).

5. The sample size is too small, with N = 2 to 3 under each condition. More mice should be recorded under each condition to strengthen the conclusions.

We increased the sample size in the revised manuscript to reach at least N = 3 animals per condition (in total, 337 RGCs from 20 animals and 557 LGN cells from 14 animals). We then performed pairwise comparisons of many tens of cells in each condition, and drew a conclusion based on the results consistent across multiple conditions. We also confirmed that these results were not due to batch effects. We thus believe that our results are highly robust, while "surprising" as the reviewer pointed out.

6. The concentrations of chlorphenamine (5 mg/kg) and ciproxifan (12 mg/kg) used are higher than those recommended in the literature, which could lead to side effects, including nonspecific binding of other receptors.
For example, large doses of chlorphenamine may cause unwanted sedation, psychomotor retardation, and blockade of cholinergic and α-adrenergic receptors. For children, the dose is calculated based on age or body weight, ranging from 0.25 mg/kg for infants to 0.20 mg/kg for older children (https://www.drugs.com/uk/chlorphenamine-10mg-ml-solution-for-injection-leaflet.html). The ciproxifan dose used was also higher than usual: doi.org/10.1523/JNEUROSCI.22-16-07272.2002 used 1.5mg/kg; https://doi.org/10.1016/j.nlm.2010.10.008 used 3mg/kg).

The concentration of the drug we used is within the range used in the past animal studies: e.g.,

- Valle-Baurista et al. (2022): chlorphenamine, 5 mg/kg, i.p.
- Frisch et al. (1997): chlorphenamine, 5 and 10mg/kg, i.p.
- Muindi et al (2015): ciproxifan, 12 mg/kg, i.p.
- Parmentier et al. (2007): ciproxifan, 1, 3, and 10 mg/kg, i.p.
- Browman et al (2004): ciproxifan, 12.5 mg/kg, i.p.
- Yegin et al. (2023): ciproxifan, 10 or 30 mg/kg, i.p.

To maximize the effects of drug application, dosage in basic science with model animals is almost always higher than the recommended dosage for humans: e.g., Warwick et al. (2024) used 10 mg/kg of Fenistil (dimetindene) to block H1 receptors in their behavioral experiments, while its recommended dosage is 0.1 mg/kg for humans (with the minimum toxic dose of 0.5 mg/kg for children; Čečrle et al., 2024). We nevertheless acknowledge the reviewer's concern on the side effects. We indeed observed some side effects, such as pupil dilation after chlorphenamine application, likely due to its anticholinergic effects (**Figure 4K**). This is why we took an alternative perturbation approach using chemogenetic tools for further supporting our results, not just relying on the pharmacological tools.

7. Brain histamine level has a circadian pattern. The manuscript should specify the time of day when recordings for each condition were performed. Were the control and experimental condition recordings consistently performed around the same period of the day?

All experiments were performed on female mice during the day time (11am/ZT4 – 4pm/ZT9; 1pm/ZT6 ± 1 hour, mean ± standard deviation; 96% of the paired recordings were conducted within 2 hours of each other), while mice were kept under the 12h-light/12h-dark cycle. Since mice are nocturnal, this means that the subject animals should have relatively low baseline histamine levels during the experiments (Takahashi et al., 2006). We, however, do not know the exact level of histamine and its fluctuations in the retina during the recordings because it depends on many factors, including an animal's behavioral and internal states, circadian rhythm, and lighting conditions (Mochizuki et al., 1992; Nowak 1993; Haas et al., 2008). We clarified these points in the revised manuscript.

8. It is surprising that applying an H1 antagonist led to larger pupil sizes and a substantial decrease in no-running periods, opposite to what would be expected since histamine is usually associated with high vigilance.

Pupil dilation is a well-known side effect of chlorphenamine due to its anticholinergic action (Fang et al., 1998). Other common side effects of this H1 antagonist include drowsiness and restlessness, leading to less or more behavior, respectively, depending on the individuals' conditions (Haas et al., 2008); and anxiolytic-like effects were also reported in mice (Miyata et al., 2011). Given complex action of pharmacology, it is thus no surprise that here we observed some individual variabilities on the behavioral outcome of the drug application (**Supplementary Figure 3A-C**).

As the reviewer pointed out, correlation between histamine levels and various behavioral measures has been widely reported (White and Rumbold, 1988; Haas et al., 2008). To the best of our knowledge, however, causality remains elusive. Moreover, as part of the central nervous system, the retina employs not just histamine but also many other neuromodulators, synthesized inside (Pourcho, 1996) or outside the retina (Koves et al., 2016; Vereczki et al., 2024). It is a future challenge to study what behavior is under the control of histamine and vice versa to clarify behavioral relevance of our findings.

Minor comment:
Please cite references for the statement: "In particular, many prey species often sleep with their eyes open, including mice."

We revised the text and included references as follows: "some species may sleep with their eyes open (Rattenborg et al., 2000; Lyamin et al., 2008, 2021; Pigarev et al., 2011), including mice (Yüzgeç et al., 2018; Karimi Abadchi et al., 2020; Turner et al., 2020; Senzai and Scanziani, 2022)."

Reviewer #3:

In the current study, Tripodi and Asari investigate how the neuromodulator histamine affects early visual processing in mice. Using pharmacological and chemogenetic tools in awake head-fixed mice, the authors found that histaminergic projections from the tuberomammillary nucleus (TMN) of the posterior hypothalamus slowed down and weaken the visual responses of retinal ganglion cells (RGCs) and cells in the lateral geniculate nucleus (LGN). After evaluating the effects of their pharmacological interventions on pupil dynamics and locomotion (running activity) and finding no clear correlations, the authors argue that the observed histamine effects likely arise from gain modulation via H1 receptors in the retina. In addition, the authors use simple computational modeling to investigate the underlying mechanism. The authors speculate that histaminergic modulation in the early visual system may be ethologically beneficial for nocturnal animals, such as mice, as this could allow for faster responses to visual threats when histamine levels are low (i.e. during less active periods).

Strengths:

This work is experimentally strong: The authors combine electrophysiological in vivo recordings with pharmacological perturbations of RGC and LGN activity, chemogenetic manipulation of histaminergic cells in the TMN, behavior as well as computational analyses to dissect the effects histamine on visual signal processing starting in the retina. In particular, the comparison between the pharmacological and chemogenetic approaches is instructive as it shows that similar manipulations of the same pathway can lead to different effects (i.e., if the release neurons are directly manipulated or if the presynaptic autoreceptors are modulated).

Major points:

Unfortunately, in its current form, the manuscript falls short in several aspects.

(1) The authors collected a very interesting and relevant dataset but the discussion of the results is weak. It almost seems as if the findings that histamine (which is expected to be high in periods of activity / <u>during day time</u>) slows and reduces activity in the early visual system surprised the authors, and they did not know how to reconcile this with expectations and earlier studies. As a result, the discussion is rather superficial. For instance, it remains unclear why mice need faster responses when they are asleep but not during their activity periods, in particular as predation seems likely whenever mice are active (and visible). How does this relate to findings in other species? The authors mention experiments in rabbits ("histamine leading to slow depolarization in LGN cells") but do not put this in context with their findings in mice.

The reported histaminergic effects on the nervous system in the literature have been diverse, species-dependent, and at times even controversial. We went through the literature once again and updated the discussion section in the revised manuscript. In particular, we would like to highlight the following points:

1) Histamine level in the retina.
The activity of central histaminergic neurons generally follows the circadian rhythm, showing high levels of activity during the active period (i.e., <u>night time</u> for nocturnal animals, such as mice) and low levels during the sleep period (Mochizuki et al., 1992; Takahashi et al., 2006; Haas et al., 2008). As

we performed our experiments during daytime (ZT4-ZT9), the retinal histamine level of the subject mice during the recordings was expected to be relatively low. This may explain why we found stronger effects by chemogenetically activating HDC+ neurons in TMN or pharmacologically blocking H3 receptors (mimicking an increase of histamine) than by administering H1 antagonists (mimicking a decrease of histamine).

These histaminergic neurons, however, show substantial variation in their endogenous activity levels depending on an animal's behavioral and internal state (Takahashi et al., 2006; Fujita et al., 2017; Michael et al., 2021). Moreover, while the mammalian retina contains a comparable level of histamine with other brain areas (around 20-140 ng/g wet tissue, i.e., about 0.1-1 μM; Nowak 1993), light stimulation of dark-adapted rabbits at night led to a substantial reduction of histamine in the retina and the optic nerve (by about 40-50%), but not in other parts of the central nervous system (Nowak 1993). This suggests that the histamine release from TMN HDC+ cells and/or the histamine metabolism are both tissue- and species-specific. Causality also remains elusive if histamine – or the activity of histaminergic neurons – itself drives a certain behavior or the other way around. It would be interesting in future studies to characterize the anatomical and physiological features of those HDC+ cells projecting to the retina and clarify behavioral relevance of our findings.

2) Histaminergic effects on the RGC/LGN physiology
Previous ex vivo studies have reported diverse histaminergic effects across all five major cell types in the retina (Vila et al., 2012; Yu et al., 2009; Yu et al., 2011; Horio et al., 2018). Primate RGCs, for instance, either increase or decrease their baseline activity, while their visual responses typically become weaker and slower after histamine bath-application (Gastinger et al 2004; Akimov et al., 2010). In contrast, rodent RGCs mostly show an increase in their baseline activity, with various changes in their visual response properties in a histamine dose-dependent manner (Gastinger et al 2004; Warwick et al., 2024). Some of these changes are interrelated: e.g., a broadening of direction-selectivity (DS) tuning in DSGCs can be explained as a natural consequence of the increased baseline activity because a decrease of the DS index (DSI) should follow by definition. For instance, let's say $R_{\mathrm{p}} = 1 + b$ and $R_{\mathrm{n}} = b$ are the responses in the preferred and null directions, respectively, with the baseline activity b. We then have $DSI = (R_{\mathrm{p}} - R_{\mathrm{n}})/(R_{\mathrm{p}} + R_{\mathrm{n}}) = 1$ with $b = 0$ whereas $DSI = 0.33$ with $b = 1$. Indeed, a reduction of DSI was reported at RGC axon terminals, caused by an increased baseline activity via arousal (Schroeder et al., 2020; Liang et al., 2020). However, the underlying circuit mechanisms of the histaminergic neuromodulations remain mostly unclear.

Here we found that histamine generally reduces and slows down RGC/LGN responses in awake mice, regardless of their functional cell-types (**Supplementary Figures 4 and 7**). We speculate that the discrepancies between our results and the previous findings are due to differences in the recording conditions, including the retinal environment. We monitored RGC activity using in vivo optic tract recordings in awake mice during day time, where the retina is exposed to various intrinsic and extrinsic neuromodulators, including histamine. In contrast, almost all previous studies have used retinal explants that should have virtually no histamine because the retina itself does not produce histamine (Gastinger et al., 1999; Greferath et al., 2009). Moreover, histamine was bath-applied at much higher concentrations (5-100 μM) to examine the histaminergic effects ex vivo than at the physiological level (<1 μM; Nowak 1993). The retinal environment was thus substantially different between previous ex vivo studies and our in vivo study.

Warwick et al. (2024) performed optic tract recordings in urethane-anesthetized mice. It has been shown, however, that RGC physiology under anesthetized conditions is rather similar to that ex vivo (e.g., lower baseline activity and slower visual responses), but not to that in the awake condition (Boissonnet et al., 2023). In particular, RGCs in awake mice exhibit high baseline activity (by ~20 Hz on average), even higher than the mean baseline activity after histamine application ex vivo (an increase by ~4 Hz on average with 5-20 μM histamine; Warwick et al., 2024). While we are unsure if this is due to the baseline retinal histamine or other factors in vivo, this clearly suggests that findings from previous ex vivo studies may not be directly applicable to the awake, more natural condition.

To our knowledge, we are the first to report histaminergic effects on dLGN in awake mice. Recordings from dLGN in cats and guinea pigs (under the anesthetized condition or brain slices; McCormick and Williamson, 1991; Uhlrich, et al., 2002) suggest that histamine leads to a slow depolarization of the relay cells, enhancing the baseline activity (unlike our results) but introducing a lag in their visual responses (like our results). Further studies are needed to address how histamine affects visual processing and perception in awake animals in tissue- and species-specific manners.

(2) A recent paper by Warwick et al. (2024) studied histaminergic feedback in the early visual system in detail and partially with similar methods like the authors. Tripodi and Asari cite the paper but fail to discuss their results in the context of the findings by Warwick et al. In view of the overlap between the two studies, it is unclear to me why this opportunity of discussing both sets of findings in a broader context was missed. Are the results consistent or where do they diverge? For instance, Warwick et al. reported specific histaminergic effects on direction-selective ganglion cells; have the authors looked into this aspect?

The revised manuscript expanded the discussion on the results reported in previous studies. Below is a brief summary with respect to Warwick et al. (2024):

1) Using the mouse retinal explants, Warwick et al. (2024) showed that histamine increased the baseline activity of RGCs. The effects were dose-dependent, and saturated at 5 μM histamine with an increased baseline firing by ~4 Hz on average. We instead found a reduced firing upon H3 antagonist application or chemogenetic activation of TMN HDC+ cells, while an enhanced firing after blocking H1 receptors. This discrepancy might be because the baseline activity of RGCs in awake mice is already high, by ~20 Hz compared to the ex vivo condition (Boissonnet et al., 2023). Alternatively, the enhancement of the baseline activity ex vivo could be an artefact of histamine overdose. Warwick et al. (2024) reported that bath-application of histamine at the physiological range (0.1-1 μM; Nowak, 1993) or that of H3 antagonists had no effect on the baseline firing ex vivo. Moreover, chemogenetic activation of HDC+ axon terminals in retinal explants led to a net reduction of firing, with ~10% of RGCs showing a significant decrease of the baseline firing while only ~1% showing an increase (Warwick et al., 2022, bioRxiv; this result was removed from their peer-reviewed publication in 2024, though). Corresponding data were not shown on their anesthetized optic tract recordings (Warwick et al., 2024). Further experiments are needed to elucidate the histaminergic effects on the baseline activity and address the discrepancies from our results, especially because these effects are species-specific and can be bi-directional (Gastinger et al 2004; Akimov et al., 2010).

2) Using two-photon calcium imaging and cell-attached recordings in isolated retinal tissues, Warwick et al. (2024) reported that histamine (20 μM) also affects RGC visual responses in a cell-type specific

manner. In contrast, we did not find substantial cell-type dependence (**Supplementary Figure 4**; LGN, **Supplementary Figure 7**). Due to technical limitations, however, here we only categorized visual response properties of RGCs/LGN cells into 6 broad groups each. In particular, our data sets were not large enough to obtain all the ~40 physiological cell-types (Baden et al., 2016; Rosón et al., 2019); and we could not target a specific cell-type or tailor visual stimuli for each cell during recordings. This is why we primarily focused on the responses to full-field stimuli to maximize the yield. Future studies should address such technical challenges and study in more detail cell-type specific effects of histamine in vivo under behaviorally more natural – thereby biologically more relevant – conditions.

3) Warwick et al (2024) reported that histamine enhances DSGC responses to faster moving stimuli in retinal explants (10 μM bath-application) and anesthetized mice (chemogenetic activation of HDC+ cells in TMN), and further showed that H1 antagonist (dimetindene, 10 mg/kg) reduces the mouse optomotor responses to moving gratings at faster temporal frequencies (5 Hz but not <2 Hz). In contrast, we found no substantial histaminergic effect on the direction- or orientation-selectivity of RGCs in awake, head-fixed mice (**Supplementary Figure 9**). Analyses on the temporal filters also indicate that the temporal frequency tuning remained largely unaffected in both RGCs (**Figure 3L**) and LGN (**Figure 2L**). Although chemogenetic activation of the TMN HDC+ neurons led to an elongation, not just lagging, of the temporal filters, we think that this goes beyond histamine, such as GABA co-release (Yu, et al., 2015), because neither H1 or H3 blockers affected their peak frequencies. Such discrepancies between the reported and our results may arise from differences in the endogenous histamine level as it depends on many factors, including an animal's behavioral and internal states and lighting conditions (Haas et al., 2008). On the one hand, mice undergoing an optomotor response may be in high attentive state with high histamine, which affects visual processing in the whole brain. On the other hand, mice under head fixation may stay relaxed, especially after habituation. More controlled studies will be needed to address the discrepancies on the histaminergic effects.

The authors speculate that histaminergic modulation in the early visual system may be ethologically beneficial for nocturnal animals, such as mice, as this could allow for faster responses to visual threats when histamine levels are low (i.e. during less active periods).

Histamine affects neural computations in many different ways, serving as one of many neuromodulators in the brain. It is thus our pure speculation that faster visual processing at low histamine levels might be beneficial for visual threat detection during less active periods. Indeed, many amphibians, reptiles, and fish lack eyelids; and mice are no exception in that they can sleep with their eyes open (Rattenborg et al., 2000; Lyamin et al., 2008, 2021; Pigarev et al., 2011; Yüzgeç et al., 2018; Karimi Abadchi et al., 2020; Turner et al., 2020; Senzai and Scanziani, 2022). It is a future challenge to further pursue behavioral relevance of the histaminergic modulation of the early visual system, starting from the retina.

(3) To better appreciate the results, it is needed to discuss histamine release and experimental conditions in more detail: Is histamine released more dependent on sleep/activity or does the day-night cycle also play a role? In this context, when were the experiments performed? Always at the same time of the day? When relative to the day-night cycle of the animals? Could the fact that the H1

antagonist had smaller effects on neural activity be explained by a low endogenous level of histamine during the experiments?

As described above (reviewer #2, major item #7), all experiments were performed during the day time (ZT4-ZT9). Since mice are nocturnal, we expect that the histamine level was generally low during the experiments. Moreover, as evidenced by low spontaneous running behaviour, the mice seemed to stay relaxed under the rig after habituation. This further supports our expectation on the low histamine level. However, histamine release depends on various factors, including the circadian rhythm, animal's behavioral and internal states, and lighting conditions (Mochizuki et al., 1992; Nowak 1993; Haas et al., 2008). Future studies should address the relationship between the activity of the retino-projecting histaminergic neurons, RGC dynamics, and an animal's behavior.

(4) The conclusion that histamine action in the retina is the main driver for the observed effects largely rests on the behavioral data. Can the authors provide supporting statistics for the pupil dynamics/locomotion data in support of their claim?

We have further analyzed the statistics and the effects of pupil size, pupil dynamics, and locomotion (**Figure 4 and Supplementary Figures 3 and 10**). For details, please see above our reply to the reviewer #1, major points 1-3. In short, we excluded the data during the running period in our analysis to minimize the side effects of locomotion. Pupil dynamics had little effect on the response latencies, whereas pupil size effects were too small to explain the histaminergic effects driven by the pharmacological or chemogenetic manipulations.

(5) It is unclear what the model adds: Specifically, it is difficult to relate the data (Fig. 4F-I) to the model predictions (Fig. 4A-E). Regarding the data: Cell types and experimental conditions are resolved in the scatter plot but then all data are fit together? The logic behind this needs to be explained.

The model provides a minimal phenomenological explanation to our main finding: i.e., gain modulation likely underlies the changes in the peak latency, but not the peak frequency. This will help form a hypothesis to clarify the underlying molecular and cellular mechanisms in future studies.

As we found no cell-type dependence in our datasets, we put all data together in our analysis. We also combined the data from different experimental conditions to assess the relationship between the two dependent variables (peak latency or frequency ratio versus nonlinearity ratio) as a result of histamine level manipulation (independent variable). In agreement with the model behavior, our data demonstrated that the more the histamine (with H1 blocker), the higher the nonlinearity ratio and the lower the peak latency ratio, whereas the less the histamine (with H3 blocker or chemogenetics), the lower the nonlinearity ratio and the higher the peak latency ratio. The revised manuscript clarified these points.

Minor points:

1. Why are the authors not showing a comparison of the data before/after saline injection (i.e. in Figs. 1 and 2)?

Population data before/after saline injection were shown in Figures 1I-L and 2G-J of the original manuscript. For clarification, we included the representative examples as well in the revised manuscript (**Figures 2F,J and 3F,J**).

2. In Suppl. Fig. 3C, all effects go in the same direction, suggesting that RF sizes shrink independent of the manipulation. In Suppl. Fig. 3E, PSAM and the H3 antagonist act differently. Please discuss.

To better characterize the spatial response properties of the recorded cells, we reperformed the RF analysis using subsampled data where the eye position was relatively stable at the resting position (**Supplementary Figures 5 and 8**). We then found no significant changes in the RF size of LGN cells or RGCs.

Differences in the outcome of the pharmacological or chemogenetic treatments from the expectations likely arose from complex side effects of the manipulations – e.g., ciproxifan has modest affinity to adrenergic receptors (Esbenshade et al., 2003) and inhibits monoamine oxidase A and B (Hagenow et al., 2017), while some HDC+ cells co-release histamine and GABA (Yu et al., 2015). It is, however, beyond the scope of this study to reconcile these results. Here we instead focused on the observations that were consistent across different conditions to draw robust conclusions (i.e., the effects of histamine on RGC/LGN visual response kinetics).

How effective is the chemogenetic activation of HDC+ cells in TMN, that is, how many histaminergic neurons in the TMN are likely to be activated? In this context, what are the endogenous levels of histamine released from the TMN to the retina?

No data is readily available to address the effectiveness of our chemogenetic manipulations besides electrophysiology. The mouse brain contains several thousand of HDC+ cells in total (Parmentier et al., 2002), but only a small fraction seems to project to the retina (Greferath et al., 2009; Lin et al., 2023; Warwick et al., 2024). We indeed found that chemogenetic manipulation of the posterior part of the dorsal (core) TMN was effective, while that of the anterior part was not ("non-TMN" group; **Figure 3 and Supplementary Figures 6 and 7**), which served as a negative control in our study. The exact number of the labelled cells projecting to the retina is unknown as we could not detect any in our histological analysis (**Supplementary Figure 2**).

As described above (major point 1), the activity of central histaminergic neurons generally follows the circadian rhythm (Mochizuki et al., 1992; Nowak 1993; Haas et al., 2008), but also fluctuates in a way dependent on an animal's behavioral and internal states as well as lighting conditions (Takahashi et al., 2006; Fujita et al., 2017; Michael et al., 2021). Moreover, these dynamics can be tissue-specific, while the histamine level in the mammalian retina is comparable to that in other brain tissues (around 0.1-1 μM; Nowak, 1993). Future, more targeted studies should address the anatomical and physiological features of those HDC+ cells projecting to the retina under different behavioral contexts.

3. What about the co-release of GABA and its potential other interactions mentioned by the authors? Could GABA effects explain the difference between the chemogenetic and H1 antagonist perturbation?

Chemogenetic activation of HDC+ cells may drive the co-release of GABA (Yu et al., 2015), while pharmacologically blocking H1 receptor should not affect the GABA level per se. We can thus safely say that the co-release of GABA by HDC+ neurons in TMN is unlikely to play a role in the observed effects. The revised manuscript clarified this point.

4. Why did the authors manipulate H1 and H3 receptors and not H2? Please explain.

To pharmacologically modulate retinal histamine levels, one can consider different types of agonists or antagonists targeting the four histamine receptor types. Here we chose H1 and H3 antagonists to effectively mimic a decrease and increase of the histamine level, respectively, based on the known effects on the retina (Gastinger et al 2004; Warwick et al., 2024) and the drug availability. We did not target H2 receptors because they are predominantly expressed on the Müller glia cells in the mouse retina (Greferath et al 2009), and pharmacologically blocking H2 receptors does not alter the effects of histamine administration on the mouse RGCs in vitro (Warwick et al., 2024). We did not study H4 because its expression was primarily in the mouse retinal pigment epithelium (Kwon et al 2024) and its role in visual responses has not been tested to our knowledge, making it difficult to have a good expectation on the outcome.

5. The functional classification of RGC and LGN types using temporal STAs obtained from responses to full-field white-noise stimuli is limited. The authors classified the responses into six broad functional groups, separated by response polarity and kinetics. However, there are other cell types found in the LGN, such suppressed-by-contrast cells, which they cannot identify using this approach. In any case, the authors perform this classification but do not make use of it. Were there any cell type-specific histaminergic effects?

We do not have enough data sets to physiologically classify and identify all the cell types reported thus far (Baden et al., 2016; Rosón et al., 2019). Furthermore, differences in the retinal physiology between in vivo and ex vivo make it difficult to apply the classification methods used in previous studies. Here we did our best to broadly categorize the response types based on the estimated temporal filters (**Supplementary Figures 4 and 7**), or the responses to moving gratings (**Supplementary Figure 9**). However, we found no obvious cell-type dependence in the effects of histamine.

6. How were the pharmacological agents applied? Systemically? What was the rationale for the selection of the concentrations of the H1 and H3 agonists/antagonists? Were the observed effects reversible?

All pharmacological reagents were injected intraperitoneally. We selected the drug concentration based on the literature (see also Reviewer #2, major item #6):

- chlorphenamine, 5 mg/kg: Frisch et al. (1997), Valle-Baurista et al. (2022).

- ciproxifan, 12 mg/kg: Muindi et al (2015), Browman et al (2004), Yegin et al. (2023).
- PSEM, 5 mg/kg: Magnus et al. (2011).

In our electrophysiology experiments, we could not keep the same cells long enough to test if the observed effects are reversible or not.

Methods: Use true typesetting for equations

Done.

Methods: Recordings in dLGN and from optic nerve are not described (only surgery). Was the same surgery used for both recordings and viral injections?

Yes, the same surgical procedure was employed in all recordings. We inserted electrodes into a target area using a robotic stereotaxic system. We described details of the methods in the revised manuscript.

line 48: "Such retinal mechanisms of neuromodulation are, however, much slower than needed to modulate retinal function to follow rapid behavioral changes." This is a bold statement, are there any references supporting this?

While no direct in vivo evidence exists to the best of our knowledge, in vitro studies suggest that retinal mechanisms of neuromodulation, such as those of dopamine or nitric oxide, are on the order of minutes or longer (Jacoby et al., 2018; Goel and Mangel, 2021). These mechanisms cannot explain neural correlates of behaviour on the order of seconds (Schroeder et al., 2020; Liang et al., 2020). We included these references in the revised manuscript.

Fig 1 & 2: Suggestions: Introduce symbols for manipulations already in (C) to (E). in 1G,H, show respective "rest" of synapse with gray outline, then the synapse and the site of action is easier to understand

Thanks for the suggestions. We updated the schematic symbols to serve more effectively as visual guides, but not to overcrowd the figures.

Suppl. Fig. 2: Indicate cell types in (A) and (C) also in color instead of only symbols.

Thanks for the suggestions. We would, however, rather like to reserve color for representing experimental conditions, rather than cell types. We thus changed the hue across the symbols to better visualize the clustering results in **Supplementary Figures 4A and 7A** in the revised manuscript.
