# Supplementary material for: The central histaminergic system slows visual processing in the retina and lateral geniculate nucleus of awake mice
Source: PLoS Biol. 2025 Nov 4;23(11):e3003406. doi: 10.1371/journal.pbio.3003406 (PMC12585013; doi:10.1371/journal.pbio.3003406)
Supplement: Attachment — Submitted filename: Response_to_reviewers_R2.pdf [file pbio.3003406.s014.pdf]

We thank again the reviewers for their constructive comments (in blue). Below are our point-by-point replies (in black).

Reviewer #1:

I’m happy with the current form. There is just one little misspelling: I believe "specific-specific" should be "species-specific" in line 545.

We corrected the typo in the revised manuscript.

Reviewer #3:

The authors did a great job in answering our questions and revising the manuscript.
We have no further comments.

Thanks.
